# Global trends in total fertility rate and its relation to national wealth, life expectancy and female education

**DOI:** 10.1186/s12889-022-13656-1

**Published:** 2022-07-14

**Authors:** Haoyue Cheng, Wenliang Luo, Shuting Si, Xing Xin, Zhicheng Peng, Haibo Zhou, Hui Liu, Yunxian Yu

**Affiliations:** 1 Department of Epidemiology & Health Statistics, School of Public Health, School of Medicine, Zhejiang University, Hangzhou, China; 2 Sir Run Run Shaw Hospital, School of Medicine, Zhejiang University, Hangzhou, China; 3 Department of Public Health and Department of Anesthesiology, the Second Affiliated Hospital of Zhejiang University School of Medicine, Hangzhou, China

**Keywords:** Total fertility rate, Economy, Health, Education, Human development index, DLNM

## Abstract

**Objectives:**

Along with the development of the times and progress of the society, the total fertility rate (TFR) markedly changed in each country. Therefore, it is critical to describe the trend of TFR and explore its influencing factors. However, previous studies did not consider the time lag and cumulative effect in the associations between the influencing factors and TFR. Thus, our study aimed to analyze the associations from a new dimension.

**Methods:**

The study was employed using national-level data from the World Bank and United Nations Development Programme. Distributed lag non-linear models with 5-year lag were used to examine the independent associations between the relevant factors and TFR.

**Results:**

The cumulative exposure-TFR curves were inverted U-shaped for log gross domestic product (GDP) per capita and life expectancy at birth, while the cumulative exposure-response curves were approximately linear for female expected years of schooling and human development index (HDI). However, it is worth noting that in the developed regions, TFR increased slightly with the high level of GDP per capita, female expected years of schooling and HDI.

**Conclusions:**

Nowadays, with the growth of GDP per capita, life expectancy at birth, female expected years of schooling and HDI, TFR are on a drastic downward trend in most regions. Besides, with the development of society, when levels of the factors continued to increase, TFR also showed a slight rebound. Therefore, governments, especially those in developing countries, should take measures to stimulate fertility and deal with a series of problems caused by declining TFR.

**Supplementary Information:**

The online version contains supplementary material available at 10.1186/s12889-022-13656-1.

## Introduction

United Nations (UN) estimated that the global population may increase from 7.8 billion in 2020 to 10.9 billion by 2100 [1]. Since a 40% population increase would have strong effects on economies, food production, environment and global climate [2, 3], figuring out the causes for population growth is critical for many aspects of international and national future planning [4]. Total fertility rate (TFR) is a key driver of the size and composition of the population [5]. TFR represents the number of children that would be born to a woman if she were to live to the end of her childbearing years and bear children in accordance with age-specific fertility rates of the specified year [6]. UN assumed that TFR in all countries would converge to near replacement level (2.1) up to 2100 [1]. However, nowadays more than half of the global population lives in regions with below-replacement fertility [7]. Lesthaeghe indicated that the world is undergoing the second demographic transition, which means long-term sub-replacement fertility [8]. There is no doubt that declining fertility is a key driver behind the rapid aging of populations worldwide [9]. Furthermore, population aging can induce a series of social problems, such as serious financial challenges to all institutions, the lack of labor and so on [10]. Therefore, it is critical to describe the trend of TFR and explore its influencing factors.

As TFR is one of the major determinants of population growth rate [1], a number of studies conducted in different countries or regions identified various influencing factors of it. Based on previous theories and data from European and other countries, Galor [11] analyzed five factors of the demographic transition and declining TFR: rising income per capita, reduced infant and child mortality, increasing requirement for human capital, decline in the gender gap, and the demands of old-age security. His study emphasized that there was a weak correlation between economic development and TFR, while investment in education was a dominating force in the decline in TFR. In Philippines, TFR is influenced by race, place of residence, educational attainment level, women’s work and marriage age [12]. Contraceptive use and economy also played important roles in explaining the fertility differences [12]. Song et al. [13] indicated that the factors related to women in Korea, such as women’s perceptions about the value of marriage and children, had more powerful influence on TFR than income, employment status and government policies. Overall, although different studies had different focuses, previous studies paid more attention to the associations between economy, education, health status and TFR, and the results were inconsistent [4, 11, 12]. Furthermore, these three aspects (economy, education, and health status) can be the criteria for assessing the strength of a country, and human development index (HDI) is a statistic composite index of the related indicators of these three aspects [14].

In addition to the differences in the above results, the analysis method is also worth noting. For example, a long-term stable GDP per capita level absolutely has a greater influence on TFR than only one year’s GDP per capita level. Meanwhile, previous studies only estimated the association between the influencing factors and TFR at the same time point, without considering the time lag and cumulative effect in the association [4, 12, 15]. In addition, previous studies assumed that the associations of the influencing factors with TFR were linear relationships, but it is obvious that there were non-linear associations between many influencing factors and TFR. Therefore, we used distributed lag non-linear models (DLNMs), a modelling framework that can simultaneously represent non-linear exposure-response dependencies and delayed effects [16, 17], to analyze the associations between the factors and TFR. This methodology is based on the definition of a ‘cross-basis’, a bi-dimensional space of functions that describes simultaneously the shape of the relationship along both the space of the predictor and the lag dimension of its occurrence [16].

Based on previous studies and data available to us, we analyzed TFR in six regions and used DLNMs to examine the associations of it with three debated factors and one comprehensive index which were presumed to be influencing factors of TFR: GDP per capita (current US$), life expectancy at birth, female expected years of schooling and human development index. We hope that the conclusions of the study can provide a theoretical basis for the government to formulate policies related to fertility and population aging.

## Methods

### Regions and countries

Previous studies indicated that countries may not be statistically independent units [4, 15]. Neighboring countries may have similarities in culture, economy, or politics, and also distant countries have economic and political ties, as well as similar health status and social norms [18]. Therefore, some countries may form clusters of similar units that are significantly different from other clusters [4]. Considering that using countries as units in statistical analysis does not meet the requirement of the independent sample, the study was analyzed at regional levels. Besides, compared with analyzing all countries together, analyzing regions separately can reduce the influence of unmeasured variables. Based on the previous study [4], we divided the countries into six regions (Western Europe and related countries, Eastern Europe, Latin America and the Caribbean, Arab States, Sub-Saharan Africa and Asia), as shown in Table S1. Because of common history and degree of economic and political ties, Eastern Europe was considered as a separate region. It is worth noting that the classification in this study only included 144 countries, excluding some small countries, island nations and regions. The main reason is that the data can’t be obtained from these countries and regions. In addition, compared with these 144 countries included in the study, the excluded countries with small populations contribute less effect on regional and world TFR, and their economy, education and health status are more vulnerable to the influence of neighboring countries. Therefore, data from the 144 countries can represent the condition of these six regions.

### Data

Data were available for 144 countries which were divided into six regions from 1960 to 2020. Country-level data on TFR, GDP per capita (current US$) and life expectancy at birth were retrieved from the World Bank Open Data (https://data.worldbank.org/) [6]. Data on female expected years of schooling and human development index were from United Nations Development Programme (http://hdr.undp.org/en/data) [19]. TFR is based on data on registered live births from vital registration systems, censuses or sample surveys. For countries without vital registration systems, TFR is generally based on extrapolations from trends observed in censuses or surveys from earlier years [6]. Although TFR is an estimated indicator, it is generally considered a reliable measure of fertility in the recent past. As the current population policies related to fertility are mainly based on female education rather than male education in some aspects, the education-related variable of the study used data for females [4, 20]. The variables in the dataset are defined as follows:

- Gross domestic product (GDP) per capita (current US$) is gross domestic product divided by midyear population [6].
- Life expectancy at birth indicates the number of years a newborn infant would live if prevailing patterns of mortality at the time of its birth were to stay the same throughout its life [6].
- Female expected years of schooling represents the number of years of schooling that a girl of school entrance age can expect to receive if prevailing patterns of age-specific enrolment rates persist throughout the child’s life [19].
- Human development index is a composite index measuring average achievement in three basic dimensions of human development—a long and healthy life, knowledge and a decent standard of living [19].

Data were complete for almost all years in high-income countries, but the majority of middle- and low-income countries had incomplete data sets due to lack of data from earlier years. In most countries, data on GDP per capita was from 1960 to 2020, TFR and life expectancy at birth were from 1960 to 2019. Since female Expected years of schooling and HDI were emerging indicators, data on them were available from 1990 to 2019. In addition, the calculation method of HDI changed in 2010 [21].

### Analytical strategies

Our analytical strategies consisted of descriptive statistics, correlations and distributed lag non-linear models (DLNMs) by region for longitudinal data analysis. Firstly, we calculated TFR and values of the relevant variables (GDP per capita, life expectancy at birth, female Expected years of schooling and HDI) for each region using mean and standard deviations (mean ± SD). The results of the world and each region were the averages of the corresponding countries, and all countries have equal weight in the analysis. Besides, due to the skewed distribution of GDP per capita, log-transformed GDP per capita was used in the analysis. Secondly, the correlations between TFR and the variables were statistically evaluated using Pearson correlation. Thirdly, since the TFR in different regions were normal distributions, we used DLNMs of gaussian distribution family to evaluate the effects of the variables on TFR. The effect of different variables on TFR was calculated separately in different models. Lag models using level of the variables of lag0 to lag5 (in years) were used to estimate the associations between the variables and TFR [22]. Natural cubic spline was used as a smoothing parameter that modeled both the nonlinear variable effect and the lagged effect. Then, the fittest model was selected by Quasi-Akaike information criterion (QAIC), based on the number of knots and position, number of degrees of freedom, and smooth function for exposure-response and lag-response function of the model (Table S2 to Table S5). The world average values for each variable were selected as the reference values. Besides, the increments of TFR and 95% confidence intervals (CIs) were estimated when the levels of the variables were low (5th quartile), middle (median), and high (95th quartile).

### Sensitivity analysis

On the basis of the above analysis, in order to eliminate the influence of some countries undergoing economic recession on the trends of GDP per capita in the corresponding regions, we conducted a separate analysis for them. In this study, we defined a country in economic recession as a country whose GDP per capita receded for at least 5 consecutive years during 10 years from the year of the economic crisis. We selected four global economic crises that began in 1979, 1990, 2000 and 2007 [23], and there were 33, 9, 1 and 20 countries included respectively. The trends of log GDP per capita, HDI and TFR in these countries were plotted to explore whether economic recession could have a different influence on TFR. In addition, as the calculation method of HDI changed in 2010, we analyzed the associations between HDI and TFR before and after 2010 respectively. Furthermore, considering that the classification of the countries in this study differed from the traditional classification, we also used United Nations geographical divisions which divided the world into five regions (Europe, Americas, Africa, Asia and Oceania) to analyze the associations between the factors and TFR (Fig. S1 to Fig. S4) [24].

All the statistical analyses were conducted using R statistical software VERSION 4.0.0 (The R Project for Statistical Computing; https://www.r-project.org). *P* < 0.05 was considered as statistically significant. The mean results were presented as the increment in TFR per unit increase of each variable.

## Results

### Trends of TFR and relevant variables

Table 1 shows descriptive statistics for TFR and the relevant variables used in our analysis by periods. The worldwide mean TFR decreased steadily from 5.29 during 1960–1969 to 2.74 during 2010–2020. Decreases in TFR occurred for all regions except Eastern Europe over the 60-year time period. The most dramatic decline in mean TFR occurred in Arab States: during 1960–1969, the Arab States’ regional mean was 7.06, higher than all the other regions; while during 2010–2020, the mean for Arab States dropped to 3.11, which was lower than the mean TFR in Sub-Saharan Africa. Sizeable declines in mean TFR were in Western Europe and related countries (42%), Eastern Europe (44%), Latin America and the Caribbean (61%) and Asia (60%), while the smallest declines occurred in Sub-Saharan Africa (29%).

**Table 1:** Descriptive statistics for relevant variables used in the study overall and by region

Variables	Periods					
	**1960–1969**	**1970–1979**	**1980–1989**	**1990–1999**	**2000–2009**	**2010–2020**
World						
Total fertility rate, mean (SD)	5.29 (1.92)	4.82 (2.09)	4.25 (2.05)	3.55 (1.88)	3.03 (1.68)	2.74 (1.39)
log GDP per capita, mean (SD)	5.84 (1.18)	6.31 (1.60)	7.09 (1.61)	7.32 (1.67)	8.06 (1.68)	8.68 (1.51)
Life expectancy at birth, mean (SD)	56.35 (12.17)	60.09 (11.30)	63.66 (10.23)	65.73 (10.56)	68.32 (10.32)	71.95 (8.43)
Female expected years of schooling, mean (SD)	-	-	-	10.26 (4.02)	11.82 (3.98)	13.36 (3.51)
Human development index, mean (SD)	-	-	-	0.62 (0.17)	0.66 (0.17)	0.71 (0.16)
Western Europe and related countries						
Total fertility rate, mean (SD)	2.89 (0.54)	2.20 (0.57)	1.83 (0.40)	1.75 (0.36)	1.72 (0.35)	1.68 (0.36)
log GDP per capita, mean (SD)	7.43 (0.54)	6.64 (2.60)	7.63 (2.56)	8.51 (2.40)	10.39 (0.63)	10.72 (0.41)
Life expectancy at birth, mean (SD)	70.88 (1.90)	72.67 (1.68)	75.02 (1.32)	77.11 (1.17)	79.39 (1.24)	81.55 (1.07)
Female expected years of schooling, mean (SD)	-	-	-	14.63 (1.82)	16.59 (1.88)	17.59 (1.93)
Human development index, mean (SD)	-	-	-	0.83 (0.04)	0.88 (0.03)	0.91 (0.03)
Eastern Europe						
Total fertility rate, mean (SD)	2.83 (1.20)	2.51 (0.80)	2.23 (0.50)	1.71 (0.45)	1.48 (0.27)	1.59 (0.20)
log GDP per capita, mean (SD)	6.04 (0.21)	6.84 (0.53)	7.36 (0.48)	7.42 (0.82)	8.31 (0.88)	9.14 (0.64)
Life expectancy at birth, mean (SD)	67.22 (4.84)	69.04 (3.42)	70.07 (2.41)	70.45 (2.37)	72.63 (2.70)	75.52 (2.43)
Female expected years of schooling, mean (SD)	-	-	-	11.83 (1.30)	13.87 (1.79)	15.62 (1.20)
Human development index, mean (SD)	-	-	-	0.71 (0.05)	0.77 (0.05)	0.82 (0.04)
Latin America and the Caribbean						
Total fertility rate, mean (SD)	5.91 (1.19)	5.03 (1.16)	4.16 (1.06)	3.39 (0.87)	2.74 (0.63)	2.33 (0.41)
log GDP per capita, mean (SD)	5.86 (0.62)	6.61 (0.73)	7.25 (0.66)	7.62 (0.77)	8.08 (0.74)	8.73 (0.70)
Life expectancy at birth, mean (SD)	57.52 (7.21)	61.77 (6.41)	65.70 (5.91)	69.08 (4.92)	71.78 (4.26)	74.01 (3.62)
Female expected years of schooling, mean (SD)	-	-	-	10.98 (1.60)	12.56 (1.80)	13.74 (2.15)
Human development index, mean (SD)	-	-	-	0.63 (0.08)	0.68 (0.08)	0.73 (0.08)
Arab States						
Total fertility rate, mean (SD)	7.06 (0.59)	6.70 (0.94)	5.79 (1.27)	4.48 (1.45)	3.54 (1.32)	3.11 (1.10)
log GDP per capita, mean (SD)	5.65 (0.87)	7.11 (1.50)	7.91 (1.37)	7.72 (1.43)	8.29 (1.39)	8.68 (1.26)
Life expectancy at birth, mean (SD)	50.70 (9.01)	57.12 (8.92)	62.61 (8.31)	66.67 (7.54)	69.37 (7.04)	71.67 (6.28)
Female expected years of schooling, mean (SD)	-	-	-	9.83 (3.28)	11.30 (3.30)	11.79 (3.29)
Human development index, mean (SD)	-	-	-	0.61 (0.14)	0.66 (0.13)	0.68 (0.13)
Sub-Saharan Africa						
Total fertility rate, mean (SD)	6.78 (0.61)	6.91 (0.61)	6.65 (0.74)	5.96 (1.05)	5.40 (1.14)	4.78 (1.02)
log GDP per capita, mean (SD)	4.88 (0.62)	5.56 (0.72)	6.14 (0.75)	6.10 (0.84)	6.41 (0.90)	7.01 (0.83)
Life expectancy at birth, mean (SD)	42.38 (5.92)	46.75 (5.99)	50.66 (5.94)	50.17 (6.15)	52.19 (4.81)	59.61 (4.63)
Female expected years of schooling, mean (SD)	-	-	-	5.95 (3.23)	7.36 (2.63)	9.60 (2.07)
Human development index, mean (SD)	-	-	-	0.40 (0.11)	0.43 (0.08)	0.51 (0.08)
Asia						
Total fertility rate, mean (SD)	5.82 (1.25)	5.01 (1.61)	4.19 (1.69)	3.26 (1.56)	2.55 (1.27)	2.32 (0.91)
log GDP per capita, mean (SD)	5.19 (0.81)	5.99 (1.24)	6.98 (1.40)	7.06 (1.64)	7.43 (1.61)	8.33 (1.40)
Life expectancy at birth, mean (SD)	54.18 (9.75)	57.92 (10.73)	62.41 (9.14)	66.10 (7.58)	69.58 (6.72)	72.92 (5.97)
Female expected years of schooling, mean (SD)	-	-	-	8.57 (2.89)	10.36 (3.00)	12.75 (2.41)
Human development index, mean (SD)	-	-	-	0.57 (0.14)	0.64 (0.13)	0.70 (0.12)

No matter in the world or in the six regions, the average levels of log GDP per capita, life expectancy at birth, female Expected years of schooling and HDI were steadily increasing year by year. These indicators were always at high levels in Western Europe and related countries while at low levels in Sub-Saharan Africa. Especially in terms of GDP per capita and female Expected years of schooling, the gaps between the two regions were nearly two-fold.

### Correlations between TFR and relevant variables

Table 2 shows the correlations between TFR and the relevant variables in the same period by region. Worldwide, life expectancy at birth (Pearson’s correlation coefficient *r* = -0.86), female expected years of schooling (*r* = -0.82) and HDI (*r* = -0.88) were most strongly correlated with TFR. There was no doubt that HDI had strong correlations with economic, health, and education indicators, as it is calculated from relevant indicators in the above-mentioned fields. In addition, female expected years of schooling was highly correlated with log GDP per capita and life expectancy at birth.

**Table 2:** Pearson correlation coefficients of the relevant variables and TFR

Variables	Total Fertility rate	log GDP per capita	Life expectancy at birth	Female expected years of schooling	Human development index
World					
Total fertility rate	1	-0.66**	-0.86**	-0.82**	-0.88**
log GDP per capita		1	0.75**	0.82**	0.86**
Life expectancy at birth			1	0.84**	0.92**
Female expected years of schooling				1	0.93**
Human development index					1
Western Europe and related countries					
Total fertility rate	1	-0.22**	-0.57**	0.11*	0.10**
log GDP per capita		1	0.64**	0.38**	0.69**
Life expectancy at birth			1	0.46**	0.73**
Female expected years of schooling				1	0.69**
Human development index					1
Eastern Europe					
Total fertility rate	1	-0.42**	-0.69**	-0.35**	-0.38**
log GDP per capita		1	0.65**	0.72**	0.84**
Life expectancy at birth			1	0.63**	0.73**
Female expected years of schooling				1	0.92**
Human development index					1
Latin America and the Caribbean					
Total fertility rate	1	-0.85**	-0.85**	-0.67**	-0.81**
log GDP per capita		1	0.86**	0.77**	0.90**
Life expectancy at birth			1	0.69**	0.85**
Female expected years of schooling				1	0.90**
Human development index					1
Arab States					
Total fertility rate	1	-0.64**	-0.81**	-0.81**	-0.78**
log GDP per capita		1	0.82**	0.71**	0.88**
Life expectancy at birth			1	0.86**	0.92**
Female expected years of schooling				1	0.92**
Human development index					1
Sub-Saharan Africa					
Total fertility rate	1	-0.73**	-0.55**	-0.81**	-0.84**
log GDP per capita		1	0.66**	0.64**	0.83**
Life expectancy at birth			1	0.59**	0.68**
Female expected years of schooling				1	0.88**
Human development index					1
Asia					
Total fertility rate	1	-0.77**	-0.85**	-0.79**	-0.77**
log GDP per capita		1	0.84**	0.82**	0.93**
Life expectancy at birth			1	0.83**	0.92**
Female expected years of schooling				1	0.92**
Human development index					1

* *P* < 0.05; ** *P* < 0.01

In different regions, the correlations between TFR and the relevant variables were quite different. Western Europe and related countries, as the region with the most developed countries, had the weakest correlations between TFR and the relevant variables (*r* ranged from − 0.57 to 0.11). In contrast, in Latin America and the Caribbean, Arab States and Asia, the strength of correlations between TFR and the variables was higher (*r* ranged from − 0.85 to -0.64). It is worth noting that regardless of the region, the correlation between TFR and life expectancy at birth was relatively high.

### TFR and GDP per capita

**Fig. 1:**
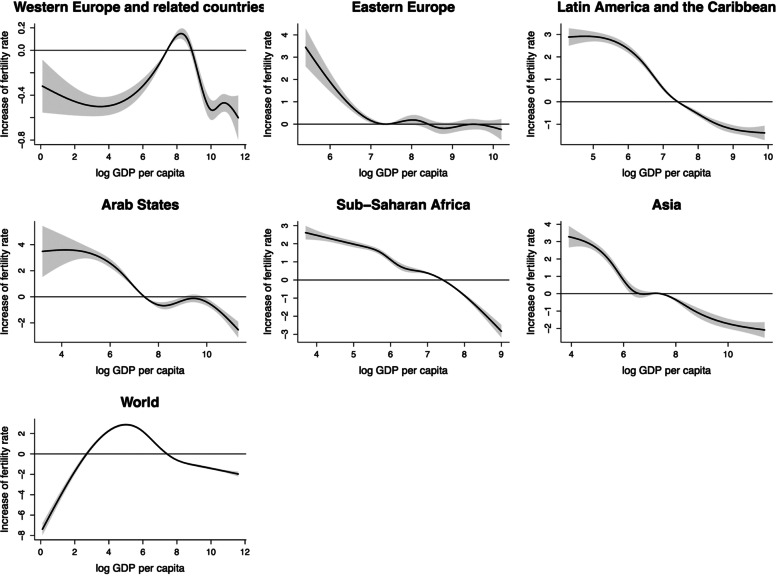
The estimated overall cumulative effects of mean log GDP per capita over 5 years on TFR

The cumulative exposure-response curves for TFR at lag0-5 are shown in Fig. 1. The cumulative exposure-response curve was approximately inverted U-shaped for log GDP per capita in the world, which meant TFR increased first and then decreased with the growth of GDP per capita. The result indicated that high GDP per capita could significantly decrease the level of TFR, and the increase of TFR was − 1.62 (95%CI: -1.74, -1.50), appearing at lag0-5 (Table S6). Within the corresponding range of GDP per capita, the trends of TFR in each region were consistent. However, TFR in Western Europe and related countries was always lower than the global average TFR, except for when log GDP per capita was between about 7 and 9. Besides, in Eastern Europe, when the TFR was higher than the average TFR around the world, it remained stable and did not change with the changes in GDP per capita.

In countries that suffered from economic crises, GDP per capita fluctuated (Fig. S5). However, TFR declined stably over the years, unaffected by fluctuations in GDP per capita. Especially in 1979–1988, GDP per capita showed a typical S-shaped fluctuation with the year, while TFR decreased linearly.

### TFR and life expectancy at birth

**Fig. 2:**
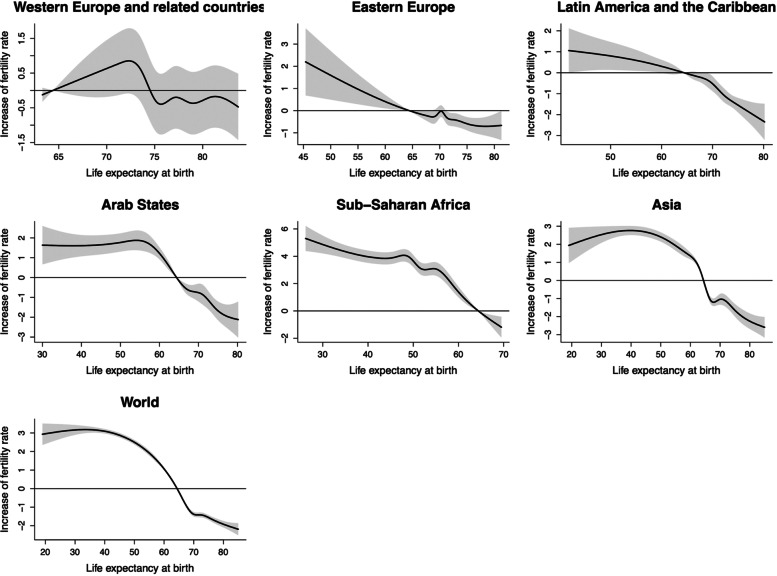
The estimated overall cumulative effects of mean life expectancy at birth over 5 years on TFR

Figure 2 illustrates the lag-specific associations of life expectancy at birth with TFR. In most regions, TFR remained stable as life expectancy at birth increased before the age of 40. Besides, it can be seen intuitively that when life expectancy at birth reached about 50 years, TFR decreased rapidly. Taking the world trend as an example, compared with the average life expectancy at birth, when it reached 43.0 years old, the increase of TFR was 2.98 (95%CI: 2.86, 3.10); while when it reached 79.9 years old, the increase of TFR was − 1.91 (95%CI: -2.09, -1.74) at lag0-5 (Table S7). However, the association of life expectancy at birth with TFR in Western Europe and related countries was not similar to the above trend, where the association was not significant. In addition, in Latin America and the Caribbean, TFR began to decline rapidly only after life expectancy at birth reached about 70 years old.

### TFR and female expected years of schooling

**Fig. 3:**
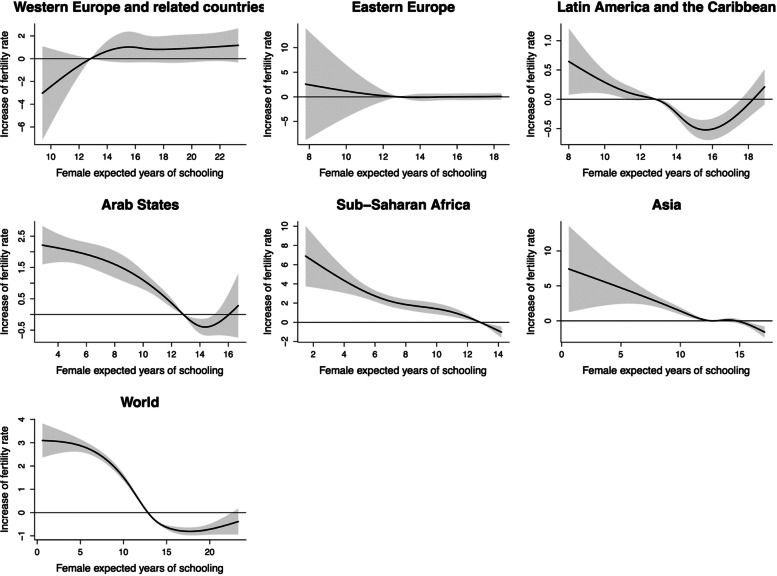
The estimated overall cumulative effects of mean female expected years of schooling over 5 years on TFR

The cumulative influence of female expected years of schooling on TFR in different regions was shown in Fig. 3. When female expected years of schooling was 12 years or less, TFR decreased stably with the increase of it. However, when female expected years of schooling reached above 15 years, TFR showed a slight rebound. It is worth noting that in developed regions (Western Europe and related countries as well as Eastern Europe), regardless of the length of female expected years of schooling, there was no significant association between it and TFR. With the high female expected years of schooling after 1990, the increases of TFR in these two regions were 0.83 (95%CI: -0.37, 2.03) and 0.08 (95%CI: -0.59, 0.75) at lag0-5, respectively (Table S8).

### TFR and HDI

**Fig. 4:**
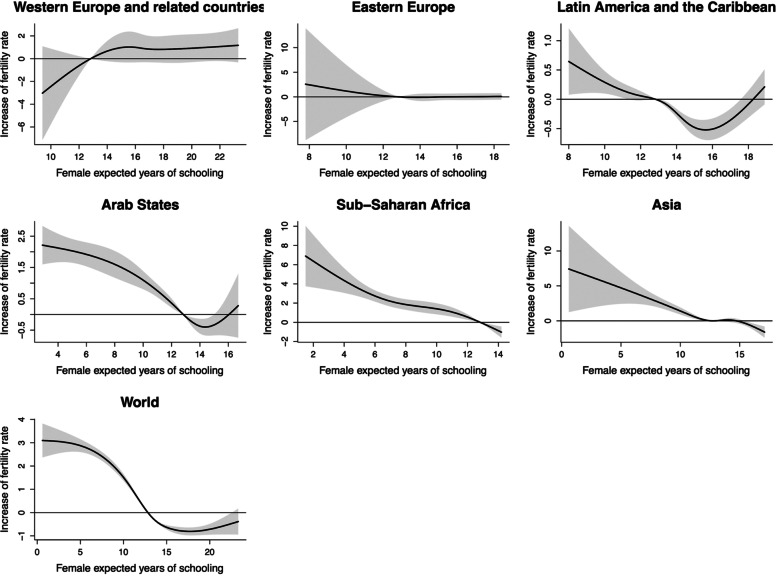
The estimated overall cumulative effects of mean human development index over 5 years on TFR

The exposure-response curves between HDI and TFR of different regions are shown in Fig. 4. TFR decreased stably along with HDI worldwide. Low-HDI could increase the level of TFR, and the increase of TFR was 3.07 (95%CI: 2.94, 3.20) at lag0-5. High-HDI also showed a negative influence on TFR, and the increase of TFR was − 0.93 (95%CI: -1.06, -0.79) (Table S9). Besides, it is worth noting that when HDI was above 0.80, TFR showed a slight rebound in the world. However, there was no correlation between TFR and HDI in Western Europe and related countries as well as Eastern Europe when HDI was above 0.75. Besides, the lag-specific associations of HDI with TFR before and after 2010 were shown in Fig. S6 and Fig. S7. These time-layered findings of associations between HDI and TFR were similar to the result above.

## Discussion

In this study, we found significant associations of TFR with GDP per capita, life expectancy at birth, female expected years of schooling and HDI. The similarity of results among regions suggested that the relationships (negative or positive) were real and fairly common. However, a number of deviations occurred in Western Europe and related countries as well as Eastern Europe. Overall, the cumulative exposure-TFR curves were inverted U-shaped for log GDP per capita and life expectancy at birth, while the cumulative exposure-response curves were approximately linear for female expected years of schooling and HDI.

To clarify factors of possible importance for TFR in different global regions, we analyzed four potential major agents that could be quantified. Over the past half century, the negative association between economic development and TFR has been one of the most unanimously accepted empirical regularities in the economic and social sciences [25–27]. In our study, long-term stable low or high GDP per capita could reduce TFR in most regions, while when GDP per capita was close to the current global average, TFR increased significantly. This result was almost in line with the above regularity. The main theory to explain the negative impact of economic development on TFR is the so-called ‘new home economic theory’. Becker et al. interpreted TFR reduction as a rational behavior of households by explaining that the increase in income induced parents to place more focus on children’s ‘quality’ rather than ‘quantity’ [28–30]. Nowadays, families are more inclined to provide better education to their children to enhance their competitiveness [31]. Based on these hypotheses, families find it optimal to have fewer children, and to provide each child with a higher level of human capital [32]. These theories together explain why TFR decline goes hand in hand with economic development.

However, some studies suggested that TFR in relation to GDP was weak, and even some researchers argued the well-established negative relationship between development and fertility had reversed [4, 32, 33]. Calderon et al. [33] confirmed that when a certain level of economic development was attained, the inverse J-shaped pattern between GDP per capita and TFR exists. In our results, the positive association of GDP per capita with TFR indeed existed in Western Europe and related countries when log GDP capita was higher than eleven. However, this association was not as obvious as in other studies. The main reason may be that the analytical method and the scope of the original data (including year and country) are different. In addition, it is worth noting that further economic development is inclined to induce TFR re-increase in the richest countries, while this increase will be small if driven by the increment in GDP per capita only [32]. And Whittington et al. [34] found that TFR was affected differently by economic development depending upon the specific sector (agriculture, manufacturing, heavy industry, and services) where growth occurred. In general, high level of GDP per capita indeed leads to the increase in TFR, but the magnitude of the increase and possible theories of the inverse relation need further studies to figure out.

The theory of ‘life history evolution’ suggests that individuals might adjust the timing of marriage and reproduction, as well as their propensity to terminate a marriage or pregnancy, in response to indicators of the locally prevailing level of life expectancy [35, 36]. Therefore, shortened timeframes lead to hastened marriage and reproduction whereas lengthier timeframes increase the probability of marital and reproductive termination. The results of our study indeed proved the theory. When life expectancy was in the range of reproductive age, TFR increased with life expectancy; otherwise, the association between these two indicators was negative. Lutz et al. [37] also confirmed that life expectancy was one of the indispensable factors which could influence TFR. He indicated that population density presented a significant factor for the levels and trends of TFR; meanwhile, population density was usually based on indicators such as life expectancy at birth [37].

Nowadays, studies about TFR in contemporary societies always focus on the relationship between education and fertility [38]. The evidence indicated that the typically negative association between women’s education and TFR was extremely stable [39–41], but the exact mechanism that leads to lower TFR with longer education is not well-known [42]. In Belgium, the postponement of fertility after 1970 is closely related to the expansion of education: compared with cohorts born in 1946–1950, 40 to 50% of the difference in cumulated fertility at age 25 in the 1951–1975 birth cohorts is attributable to rising educational levels [41]. However, our study found that the negative association of women’s education with TFR only existed when the expected years of schooling was not long enough. Some other studies also confirmed our results. Jalovaara et al. [43] concluded that in certain higher-income countries, the negative relationship between them was diminishing in cohorts. Nisen et al. [38] further put forward the contention that there was higher TFR in high-educated women compared to that in medium- (or low-) educated women in more developed regions. This phenomenon indeed appeared in our results, especially in Latin America and the Caribbean as well as Arab States. Nevertheless, the theories about the above phenomenon remain unclear yet. A possible explanation is that although highly educated women have larger earnings losses from family leaves than low-educated women, their remaining income may still be more than sufficient to maintain the family’s previous level of living [43, 44]. Therefore, more studies are needed to explore and explain the current association between education and fertility.

First calculated in 1990, HDI serves as a frame of reference for both social and economic development. HDI measures the average achievements of a country in a single statistic by combining indicators of health, knowledge, and standard of living [45]. Studies found that the association between HDI and TFR was similar to that between GDP per capita and TFR [26, 46]. In recent years, the association of HDI with TFR is negative for HDI levels below the range of 0.85–0.9. However, as the HDI is close to levels above about 0.9, the HDI-TFR association reverses to a positive relationship: higher levels of HDI are associated with higher levels of TFR [46]. In our study, the inverse association was not obvious, probably because the regions were not divided according to the level of HDI. Given the heterogeneity of institutional, cultural and policy contexts across countries, further research is required to investigate the different mechanisms that may underlie the reversal in the association between HDI and TFR [46]. However, based on the previous and our studies, as a comprehensive indicator, HDI is currently a relatively representative indicator that can be used to assess the relationship between the overall development level of a country and TFR.

Overall, there is no doubt that numerous countries are experiencing extremely rapid fertility decline nowadays. More and more countries are undergoing the second demographic transition. The rise of cohabitation, the progress in women’s rights, and advanced concepts were structural features causing fertility postponement [8, 47]. Although TFR shows a slight rebound when GDP per capita, female expected years of schooling or HDI reaches a certain level, for some developing countries, the adverse effects of the decline in fertility will have been very serious by then. Take China as an example, evidence from the 2020 Census suggests that TFR may have fallen to 1.3 in China [48]. And the continuing sluggish TFR has deepened the degree of population aging, which inevitably gives rise to a host of socioeconomic challenges [49]. Population aging induces many challenges and concerns about the pace of future economic growth, the operation and financial integrity of health care and pension systems, the well-being of the elderly and labor-force shortages [50, 51]. Therefore, governments, especially those already faced with the declining TFR, should formulate relevant policies to promote fertility as soon as possible, because the situation of declining TFR will continue for a long time. Our study found that increasing female expected years of schooling high enough was the most effective way to promote TFR. Women’s consciousness about the value of marriage and children affected childbirths, which meant that it may be quite difficult for women to give up their careers and choose childbirth without social support or adequate compensation in modern society [13]. Therefore, social support systems and relative policies should be pursued to help women balance their career and child rearing, such as paid parental leave, gender equality in parental leave and so on [52]. However, it cannot be ignored that TFR is still too high in some poor developing countries, which is above the replacement level, such as Niger, Uganda, and Afghanistan. High fertility is leading to profound local environmental pressures, including water stress, land degradation, and decreasing farm sizes, which are worsening the serious economic challenges these countries face [53]. High fertility also forces children to suffer from woeful under-investments in education, health and nutrition. Therefore, for these countries, how to reduce fertility is the primary consideration. Overall, different governments can choose appropriate countermeasures according to national conditions.

Our study assessed the influence of three debated factors and one comprehensive index on TFR, and further analyzed the associations in different regions. However, it has several limitations. First, although considering the influence of the levels of each factor in the previous five years on the subsequent TFR, the actual causal association between them is not clear. For example, some studies indicated that with social and economic development, lower TFR may promote economic development, rather than the other way around [54, 55]. Second, when analyzing the association between the factors and TFR, other confounding variables were not controlled. And the strength of correlations between TFR and these variables was high. Therefore, the associations between TFR and the variables were not guaranteed to be correct, and a cautious approach in interpreting the results was warranted. Third, there was a possibility that unique TFR in some countries had been masked by grouping the countries. Therefore, the results tended to represent average associations between the relevant factors and TFR, ignoring the specificity of each country. Forth, owing to lack of related data in some countries and regions, we did not include these countries and regions in the analysis. Therefore, some results might not represent the real-world level. Fifth, since our data was obtained from public databases, some indicators (such as religion, contraceptive use, family planning programs and so on) were not included in this analysis. However, it is undeniable that these indicators do have influence on TFR [4].

In general, our study is the first one to take the time lag and cumulative effect into account when assessing the relationship between GDP per capita, life expectancy at birth, female expected years of schooling, HDI and TFR. And the non-linear associations were revealed intuitively. The results of our study confirmed the associations between the relevant factors and TFR found in the previous studies, and added another dimension to examine the relationship. In addition, based on the second demographic transition theory, further studies should be conducted to explore the associations between the postmodern ideas/attitudes and TFR rather than being limited to objective country-level indicators.

## Conclusions

Over the last decades, in many countries TFR dropped drastically. The trend in TFR was related to various factors. The cumulative exposure-TFR curves were inverted U-shaped for log GDP per capita and life expectancy at birth, while the cumulative exposure-response curves were approximately linear for female expected years of schooling and HDI. These stable associations existed in all regions except for Western Europe and related countries as well as Eastern Europe. In the above two regions, the TFR increased slightly with the high GDP per capita, female expected years of schooling and HDI. These associations indicated that with the development of society, when levels of the factors continued to increase, TFR also showed a slight rebound. Taking into account the situation of declining TFR will continue for a long time, governments, especially those already faced with the continuously declining TFR, should take countermeasures to stimulate fertility and alleviate the pressure resulting from low TFR.

## Supplementary Information

**Additional file 1.**

## Data Availability

The datasets generated and/or analyzed during the current study are available in the World Bank Open Data (https://data.worldbank.org/) and United Nations Development Programme (http://hdr.undp.org/en/data).

## Abbreviations

CI: Confidence Interval
DLNM: Distributed Lag Non-linear Model
GDP: Gross Domestic Product
HDI: Human Development Index
TFR: Total Fertility Rate
UN: United Nations
