# Supplementary material for: Global trends in total fertility rate and its relation to national wealth, life expectancy and female education
Source: BMC Public Health. 2022 Jul 14;22:1346. doi: 10.1186/s12889-022-13656-1 (PMC9284852; doi:10.1186/s12889-022-13656-1)
Supplement: Supplementary file 1 — Additional file 1. [file 12889_2022_13656_MOESM1_ESM.docx]

| **Table S1** Six regions and 144 countries included in the analysis | | | | |
| --- | --- | --- | --- | --- |
| Region | Country | | | |
| Western Europe and related countries | ﻿Portugal | ﻿Spain | ﻿Greece | Cyprus |
|  | Malta | Germany | Italy | Austria |
|  | Switzerland | Luxembourg | Canada | Denmark |
|  | Netherlands | ﻿Finland | ﻿Belgium | Norway |
|  | United States | United Kingdom | ﻿Australia | Sweden |
|  | France | Iceland | Ireland | New Zealand |
|  | ﻿Israel |  |  |  |
| Eastern Europe | Bosnia and Herzegovina | Hungary | Poland | Slovak Republic |
|  | Czech Republic | Romania | Croatia | Ukraine |
|  | Latvia | Bulgaria | Slovenia | Estonia |
|  | Serbia | Lithuania | Belarus | Armenia |
|  | Montenegro | Albania | Georgia | Turkey |
|  | Russian Federation |  |  |  |
| Latin America and the Caribbean | Brazil | Trinidad and Tobago | Chile | Costa Rica |
|  | Colombia | Uruguay | Jamaica | El Salvador |
|  | Mexico | ﻿Nicaragua | Argentina | Venezuela, RB |
|  | Peru | Dominican Republic | Ecuador | ﻿Paraguay |
|  | Guyana | Panama | Belize | Honduras |
|  | Bolivia | Haiti | Guatemala |  |
| Arab States | Lebanon | United Arab Emirates | Qatar | Kuwait |
|  | Bahrain | Tunisia | Libya | Morocco |
|  | Saudi Arabia | Algeria | Egypt, Arab Rep. | Jordan |
|  | Yemen, Rep. | Iraq | Comoros | Sudan |
|  | South Sudan | Mauritania |  |  |
| ﻿Sub-Saharan Africa | South Africa | Botswana | Eswatini | Namibia |
|  | Zimbabwe | Kenya | Ghana | ﻿Rwanda |
|  | Madagascar | Togo | Liberia | Sierra Leone |
|  | Congo, Rep. | Malawi | Cameroon | Senegal |
|  | Central African Republic | Guinea | Cote d'Ivoire | Zambia |
|  | Benin | Tanzania | ﻿Mozambique | Burkina Faso |
|  | Nigeria | Uganda | Burundi | Chad |
|  | Mali | Congo, Dem. Rep. | Niger |  |
| Asia | Hong Kong SAR, China | Singapore | Korea, Rep. | Japan |
|  | Thailand | Iran, Islamic Rep. | Vietnam | Azerbaijan |
|  | Malaysia | Bangladesh | Myanmar | Nepal |
|  | Uzbekistan | India | Indonesia | Cambodia |
|  | Kazakhstan | ﻿Mongolia | Lao PDR | Philippines |
|  | Kyrgyz Republic | Tajikistan | Pakistan | Afghanistan |
|  | Macao SAR, China | China |  |  |

| **Table S2** Model parameter selection for different GDP per capita (exposure-response) and lag-response functions | | | | |
| --- | --- | --- | --- | --- |
| Model | Exposure-response | Lag-response | Total df | QAIC |
| Model 1 | natural cubic spline^a^ | natural cubic spline^g^ | 2 | 24333.3 |
| Model 2 | natural cubic spline^b^ | natural cubic spline^g^ | 4 | 24065 |
| Model 3 | natural cubic spline^c^ | natural cubic spline^g^ | 6 | 22444.2 |
| Model 4 | natural cubic spline^d^ | natural cubic spline^g^ | 8 | 22192 |
| Model 5 | natural cubic spline^e^ | natural cubic spline^g^ | 10 | 22181.5 |
| Model 6 | natural cubic spline^f^ | natural cubic spline^g^ | 12 | 22188.2 |
|  |  |  |  |  |
| Model 7 | natural cubic spline^a^ | natural cubic spline^h^ | 3 | 24255.5 |
| Model 8 | natural cubic spline^b^ | natural cubic spline^h^ | 6 | 23985.5 |
| Model 9 | natural cubic spline^c^ | natural cubic spline^h^ | 9 | 22316.1 |
| Model 10 | natural cubic spline^d^ | natural cubic spline^h^ | 12 | 22046.6 |
| Model 11 | natural cubic spline^e^ | natural cubic spline^h^ | 15 | 22038 |
| Model 12 | natural cubic spline^f^ | natural cubic spline^h^ | 18 | 22045.9 |
|  |  |  |  |  |
| Model 13 | natural cubic spline^a^ | natural cubic spline^i^ | 4 | 24232.9 |
| Model 14 | natural cubic spline^b^ | natural cubic spline^i^ | 8 | 23956.2 |
| Model 15 | natural cubic spline^c^ | natural cubic spline^i^ | 12 | 22279.5 |
| Model 16 | natural cubic spline^d^ | natural cubic spline^i^ | 16 | 22015.8 |
| Model 17 | natural cubic spline^e^ | natural cubic spline^i^ | 20 | 22008.9 |
| Model 18 | natural cubic spline^f^ | natural cubic spline^i^ | 24 | 22019 |
|  |  |  |  |  |
| Model 19 | natural cubic spline^a^ | natural cubic spline^j^ | 5 | 24229.1 |
| Model 20 | natural cubic spline^b^ | natural cubic spline^j^ | 10 | 23953.9 |
| Model 21 | natural cubic spline^c^ | natural cubic spline^j^ | 15 | 22275.5 |
| Model 22 | natural cubic spline^d^ | natural cubic spline^j^ | 20 | 22012.3 |
| Model 23 | natural cubic spline^e^ | natural cubic spline^j^ | 25 | **22007.4** |
| Model 24 | natural cubic spline^f^ | natural cubic spline^j^ | 30 | 22019.5 |
|  |  |  |  |  |
| Model 25 | natural cubic spline^a^ | natural cubic spline^k^ | 6 | 24230.1 |
| Model 26 | natural cubic spline^b^ | natural cubic spline^k^ | 12 | 23956.7 |
| Model 27 | natural cubic spline^c^ | natural cubic spline^k^ | 18 | 22279.7 |
| Model 28 | natural cubic spline^d^ | natural cubic spline^k^ | 24 | 22018.7 |
| Model 29 | natural cubic spline^e^ | natural cubic spline^k^ | 30 | 22015.8 |
| Model 30 | natural cubic spline^f^ | natural cubic spline^k^ | 36 | 22029.9 |
| ^a^ natural cubic spline with 0 knots; ^b^ natural cubic spline with 1 knots placed at equal distance of GDP per capita; ^c^ natural cubic spline with 2 knots placed at equal distance of GDP per capita; ^d^ natural cubic spline with 3 knots placed at equal distance of GDP per capita; ^e^ natural cubic spline with 4 knots placed at equal distance of GDP per capita; ^f^ natural cubic spline with 5 knots placed at equal distance of GDP per capita; ^g^ natural cubic spline with 0 knots; ^h^ natural cubic spline with 1 knots placed at equal distance of lag; ^i^ natural cubic spline with 2 knots placed at equal distance of lag; ^j^ natural cubic spline with 3 knots placed at equal distance of lag; ^k^ natural cubic spline with 4 knots placed at equal distance of lag. | | | | |

| **Table S3** Model parameter selection for different life expectancy at birth (exposure-response) and lag-response functions | | | | |
| --- | --- | --- | --- | --- |
| Model | Exposure-response | Lag-response | Total df | QAIC |
| Model 1 | natural cubic spline^a^ | natural cubic spline^i^ | 2 | 22049 |
| Model 2 | natural cubic spline^b^ | natural cubic spline^i^ | 4 | 21745.4 |
| Model 3 | natural cubic spline^c^ | natural cubic spline^i^ | 6 | 20625.2 |
| Model 4 | natural cubic spline^d^ | natural cubic spline^i^ | 8 | 20634.9 |
| Model 5 | natural cubic spline^e^ | natural cubic spline^i^ | 10 | 20555.4 |
| Model 6 | natural cubic spline^f^ | natural cubic spline^i^ | 12 | 20551.1 |
| Model 7 | natural cubic spline^g^ | natural cubic spline^i^ | 14 | 20515.1 |
| Model 8 | natural cubic spline^h^ | natural cubic spline^i^ | 16 | 20520.3 |
|  |  |  |  |  |
| Model 9 | natural cubic spline^a^ | natural cubic spline^j^ | 3 | 21759.6 |
| Model 10 | natural cubic spline^b^ | natural cubic spline^j^ | 6 | 21529.8 |
| Model 11 | natural cubic spline^c^ | natural cubic spline^j^ | 9 | 20383 |
| Model 12 | natural cubic spline^d^ | natural cubic spline^j^ | 12 | 20353.4 |
| Model 13 | natural cubic spline^e^ | natural cubic spline^j^ | 15 | 20311.6 |
| Model 14 | natural cubic spline^f^ | natural cubic spline^j^ | 18 | 20284.4 |
| Model 15 | natural cubic spline^g^ | natural cubic spline^j^ | 21 | 20264.3 |
| Model 16 | natural cubic spline^h^ | natural cubic spline^j^ | 24 | 20272.6 |
|  |  |  |  |  |
| Model 17 | natural cubic spline^a^ | natural cubic spline^k^ | 4 | 21746.6 |
| Model 18 | natural cubic spline^b^ | natural cubic spline^k^ | 8 | 21509.7 |
| Model 19 | natural cubic spline^c^ | natural cubic spline^k^ | 12 | 20378.8 |
| Model 20 | natural cubic spline^d^ | natural cubic spline^k^ | 16 | 20334.7 |
| Model 21 | natural cubic spline^e^ | natural cubic spline^k^ | 20 | 20296 |
| Model 22 | natural cubic spline^f^ | natural cubic spline^k^ | 24 | 20273 |
| Model 23 | natural cubic spline^g^ | natural cubic spline^k^ | 28 | 20253.3 |
| Model 24 | natural cubic spline^h^ | natural cubic spline^k^ | 32 | 20264.3 |
|  |  |  |  |  |
| Model 25 | natural cubic spline^a^ | natural cubic spline^l^ | 5 | 21743.1 |
| Model 26 | natural cubic spline^b^ | natural cubic spline^l^ | 10 | 21489.8 |
| Model 27 | natural cubic spline^c^ | natural cubic spline^l^ | 15 | 20369.6 |
| Model 28 | natural cubic spline^d^ | natural cubic spline^l^ | 20 | 20324.1 |
| Model 29 | natural cubic spline^e^ | natural cubic spline^l^ | 25 | 20261.5 |
| Model 30 | natural cubic spline^f^ | natural cubic spline^l^ | 30 | 20225.3 |
| Model 31 | natural cubic spline^g^ | natural cubic spline^l^ | 35 | **20209.6** |
| Model 32 | natural cubic spline^h^ | natural cubic spline^l^ | 40 | 20221.6 |
|  |  |  |  |  |
| Model 33 | natural cubic spline^a^ | natural cubic spline^m^ | 6 | 21744.9 |
| Model 34 | natural cubic spline^b^ | natural cubic spline^m^ | 12 | 21493.7 |
| Model 35 | natural cubic spline^c^ | natural cubic spline^m^ | 18 | 20375.5 |
| Model 36 | natural cubic spline^d^ | natural cubic spline^m^ | 24 | 20331.7 |
| Model 37 | natural cubic spline^e^ | natural cubic spline^m^ | 30 | 20270.8 |
| Model 38 | natural cubic spline^f^ | natural cubic spline^m^ | 36 | 20236.1 |
| Model 39 | natural cubic spline^g^ | natural cubic spline^m^ | 42 | 20218.6 |
| Model 40 | natural cubic spline^h^ | natural cubic spline^m^ | 48 | 20232.5 |
| ^a^ natural cubic spline with 0 knots; ^b^ natural cubic spline with 1 knots placed at equal distance of life expectancy at birth; ^c^ natural cubic spline with 2 knots placed at equal distance of life expectancy at birth; ^d^ natural cubic spline with 3 knots placed at equal distance of life expectancy at birth; ^e^ natural cubic spline with 4 knots placed at equal distance of life expectancy at birth; ^f^ natural cubic spline with 5 knots placed at equal distance of life expectancy at birth; ^g^ natural cubic spline with 6 knots placed at equal distance of life expectancy at birth; ^h^ natural cubic spline with 7 knots placed at equal distance of life expectancy at birth; ^i^ natural cubic spline with 0 knots; ^j^ natural cubic spline with 1 knots placed at equal distance of lag; ^k^ natural cubic spline with 2 knots placed at equal distance of lag; ^l^ natural cubic spline with 3 knots placed at equal distance of lag; ^m^ natural cubic spline with 4 knots placed at equal distance of lag. | | | | |

| **Table S4** Model parameter selection for different female expected years of schooling (exposure-response) and lag-response functions | | | | |
| --- | --- | --- | --- | --- |
| Model | Exposure-response | Lag-response | Total df | QAIC |
| Model 1 | natural cubic spline^a^ | natural cubic spline^g^ | 2 | 1614.2 |
| Model 2 | natural cubic spline^b^ | natural cubic spline^g^ | 4 | 1475.7 |
| Model 3 | natural cubic spline^c^ | natural cubic spline^g^ | 6 | 1391.8 |
| Model 4 | natural cubic spline^d^ | natural cubic spline^g^ | 8 | 1378.4 |
| Model 5 | natural cubic spline^e^ | natural cubic spline^g^ | 10 | 1382.4 |
| Model 6 | natural cubic spline^f^ | natural cubic spline^g^ | 12 | 1381 |
|  |  |  |  |  |
| Model 7 | natural cubic spline^a^ | natural cubic spline^h^ | 3 | 1606 |
| Model 8 | natural cubic spline^b^ | natural cubic spline^h^ | 6 | 1460.6 |
| Model 9 | natural cubic spline^c^ | natural cubic spline^h^ | 9 | 1377.9 |
| Model 10 | natural cubic spline^d^ | natural cubic spline^h^ | 12 | 1361.9 |
| Model 11 | natural cubic spline^e^ | natural cubic spline^h^ | 15 | 1367.5 |
| Model 12 | natural cubic spline^f^ | natural cubic spline^h^ | 18 | 1368.4 |
|  |  |  |  |  |
| Model 13 | natural cubic spline^a^ | natural cubic spline^i^ | 4 | 1608 |
| Model 14 | natural cubic spline^b^ | natural cubic spline^i^ | 8 | 1461.5 |
| Model 15 | natural cubic spline^c^ | natural cubic spline^i^ | 12 | 1379.1 |
| Model 16 | natural cubic spline^d^ | natural cubic spline^i^ | 16 | 1365.3 |
| Model 17 | natural cubic spline^e^ | natural cubic spline^i^ | 20 | 1372.5 |
| Model 18 | natural cubic spline^f^ | natural cubic spline^i^ | 24 | 1375.8 |
|  |  |  |  |  |
| Model 19 | natural cubic spline^a^ | natural cubic spline^j^ | 5 | 1609.5 |
| Model 20 | natural cubic spline^b^ | natural cubic spline^j^ | 10 | 1465.1 |
| Model 21 | natural cubic spline^c^ | natural cubic spline^j^ | 15 | 1384.3 |
| Model 22 | natural cubic spline^d^ | natural cubic spline^j^ | 20 | **1372.1** |
| Model 23 | natural cubic spline^e^ | natural cubic spline^j^ | 25 | 1381.2 |
| Model 24 | natural cubic spline^f^ | natural cubic spline^j^ | 30 | 1385.4 |
|  |  |  |  |  |
| Model 25 | natural cubic spline^a^ | natural cubic spline^k^ | 6 | 1611.2 |
| Model 26 | natural cubic spline^b^ | natural cubic spline^k^ | 12 | 1469 |
| Model 27 | natural cubic spline^c^ | natural cubic spline^k^ | 18 | 1390.2 |
| Model 28 | natural cubic spline^d^ | natural cubic spline^k^ | 24 | 1378.5 |
| Model 29 | natural cubic spline^e^ | natural cubic spline^k^ | 30 | 1389.6 |
| Model 30 | natural cubic spline^f^ | natural cubic spline^k^ | 36 | 1394.6 |
| ^a^ natural cubic spline with 0 knots; ^b^ natural cubic spline with 1 knots placed at equal distance of female expected years of schooling; ^c^ natural cubic spline with 2 knots placed at equal distance of female expected years of schooling; ^d^ natural cubic spline with 3 knots placed at equal distance of female expected years of schooling; ^e^ natural cubic spline with 4 knots placed at equal distance of female expected years of schooling; ^f^ natural cubic spline with 5 knots placed at equal distance of female expected years of schooling; ^g^ natural cubic spline with 0 knots; ^h^ natural cubic spline with 1 knots placed at equal distance of lag; ^i^ natural cubic spline with 2 knots placed at equal distance of lag; ^j^ natural cubic spline with 3 knots placed at equal distance of lag; ^k^ natural cubic spline with 4 knots placed at equal distance of lag. | | | | |

| **Table S5** Model parameter selection for different HDI (exposure-response) and lag-response functions | | | | |
| --- | --- | --- | --- | --- |
| Model | Exposure-response | Lag-response | Total df | QAIC |
| Model 1 | natural cubic spline^a^ | natural cubic spline^g^ | 2 | 7875.2 |
| Model 2 | natural cubic spline^b^ | natural cubic spline^g^ | 4 | 6982.5 |
| Model 3 | natural cubic spline^c^ | natural cubic spline^g^ | 6 | 6961.3 |
| Model 4 | natural cubic spline^d^ | natural cubic spline^g^ | 8 | 6934.2 |
| Model 5 | natural cubic spline^e^ | natural cubic spline^g^ | 10 | 6931.8 |
| Model 6 | natural cubic spline^f^ | natural cubic spline^g^ | 12 | 6939.4 |
|  |  |  |  |  |
| Model 7 | natural cubic spline^a^ | natural cubic spline^h^ | 3 | 7832 |
| Model 8 | natural cubic spline^b^ | natural cubic spline^h^ | 6 | 6914.4 |
| Model 9 | natural cubic spline^c^ | natural cubic spline^h^ | 9 | 6880.2 |
| Model 10 | natural cubic spline^d^ | natural cubic spline^h^ | 12 | 6857.4 |
| Model 11 | natural cubic spline^e^ | natural cubic spline^h^ | 15 | 6859.6 |
| Model 12 | natural cubic spline^f^ | natural cubic spline^h^ | 18 | 6865.8 |
|  |  |  |  |  |
| Model 13 | natural cubic spline^a^ | natural cubic spline^i^ | 4 | 7830.1 |
| Model 14 | natural cubic spline^b^ | natural cubic spline^i^ | 8 | 6914.1 |
| Model 15 | natural cubic spline^c^ | natural cubic spline^i^ | 12 | 6880 |
| Model 16 | natural cubic spline^d^ | natural cubic spline^i^ | 16 | 6858.3 |
| Model 17 | natural cubic spline^e^ | natural cubic spline^i^ | 20 | 6862.4 |
| Model 18 | natural cubic spline^f^ | natural cubic spline^i^ | 24 | 6870 |
|  |  |  |  |  |
| Model 19 | natural cubic spline^a^ | natural cubic spline^j^ | 5 | 7831.2 |
| Model 20 | natural cubic spline^b^ | natural cubic spline^j^ | 10 | 6916.9 |
| Model 21 | natural cubic spline^c^ | natural cubic spline^j^ | 15 | 6884.3 |
| Model 22 | natural cubic spline^d^ | natural cubic spline^j^ | 20 | **6864.2** |
| Model 23 | natural cubic spline^e^ | natural cubic spline^j^ | 25 | 6869.9 |
| Model 24 | natural cubic spline^f^ | natural cubic spline^j^ | 30 | 6879.6 |
|  |  |  |  |  |
| Model 25 | natural cubic spline^a^ | natural cubic spline^k^ | 6 | 7833 |
| Model 26 | natural cubic spline^b^ | natural cubic spline^k^ | 12 | 6920.7 |
| Model 27 | natural cubic spline^c^ | natural cubic spline^k^ | 18 | 6890.1 |
| Model 28 | natural cubic spline^d^ | natural cubic spline^k^ | 24 | 6871.9 |
| Model 29 | natural cubic spline^e^ | natural cubic spline^k^ | 30 | 6879.7 |
| Model 30 | natural cubic spline^f^ | natural cubic spline^k^ | 36 | 6891.4 |
| ^a^ natural cubic spline with 0 knots; ^b^ natural cubic spline with 1 knots placed at equal distance of HDI; ^c^ natural cubic spline with 2 knots placed at equal distance of HDI; ^d^ natural cubic spline with 3 knots placed at equal distance of HDI; ^e^ natural cubic spline with 4 knots placed at equal distance of HDI; ^f^ natural cubic spline with 5 knots placed at equal distance of HDI; ^g^ natural cubic spline with 0 knots; ^h^ natural cubic spline with 1 knots placed at equal distance of lag; ^i^ natural cubic spline with 2 knots placed at equal distance of lag; ^j^ natural cubic spline with 3 knots placed at equal distance of lag; ^k^ natural cubic spline with 4 knots placed at equal distance of lag. | | | | |

| **Table S6** The effects of log GDP per capita changes (5, 50, and 95% percentiles) on TFR at lag0-5 years (reference = 7.4) | | | |
| --- | --- | --- | --- |
| Regions | log GDP per capita | | |
|  | Low (4.8) | Median (7.3) | High (10.6) |
| Increase of fertility rate (95% CI) | | | |
| Western Europe and related countries | **-0.46(-0.54, -0.38)** | **-0.03(-0.04, -0.03)** | **-0.48(-0.56, -0.40)** |
| Eastern Europe | - | 0.001(-0.04, 0.04) | - |
| Latin America and the Caribbean | **2.92(2.66, 3.17)** | **0.14(0.10, 0.17)** | - |
| Arab States | **3.50(2.93, 4.08)** | **0.20(0.16, 0.23)** | **-1.22(-1.54, -0.90)** |
| Sub-Saharan Africa | **2.08(1.90, 2.25)** | **0.13(0.12, 0.14)** | - |
| Asia | **2.79(2.55, 3.04)** | 0.02(-0.001, 0.05) | **-1.91(-2.21, -1.61)** |
| World | **2.84(2.72, 2.96)** | **0.16(0.14, 0.17)** | **-1.62(-1.74, -1.50)** |

| **Table S7** The effects of life expectancy at birth changes (5, 50, and 95% percentiles) on TFR at lag0-5 years (reference = 64.4) | | | |
| --- | --- | --- | --- |
| Regions | Life expectancy at birth (total) | | |
|  | Low (43.0) | Median (68.0) | High (79.9) |
| Increase of fertility rate (95% CI) | | | |
| Western Europe and related countries | - | 0.41(-0.19, 1.00) | -0.30(-1.20, 0.60) |
| Eastern Europe | - | -0.26(-0.52, 0.004) | **-0.70(-1.22, -0.17)** |
| Latin America and the Caribbean | **1.02(0.04, 2.01)** | -0.25(-0.50, 0.01) | **-2.30(-3.13, -1.47)** |
| Arab States | **1.63(1.13, 2.13)** | **-0.68(-0.98, -0.38)** | **-2.11(-2.98, -1.24)** |
| Sub-Saharan Africa | **3.85(3.41, 4.29)** | **-0.86(-1.39, -0.34)** | - |
| Asia | **2.73(2.47, 2.98)** | **-1.20(-1.42, -0.99)** | **-2.25(-2.68, -1.82)** |
| World | **2.98(2.86, 3.10)** | **-1.05(-1.15, -0.96)** | **-1.91(-2.09, -1.74)** |

| **Table S8** The effects of female expected years of schooling changes (5, 50, and 95% percentiles) on TFR at lag0-5 years (reference = 12.8) | | | |
| --- | --- | --- | --- |
| Regions | Female expected years of schooling | | |
|  | Low (6.0) | Middle (13.3) | High (18.4) |
| Increase of fertility rate (95% CI) | | | |
| Western Europe and related countries | - | 0.30(-0.10, 0.71) | 0.83(-0.37, 2.03) |
| Eastern Europe | - | -0.08(-0.50, 0.35) | 0.08(-0.59, 0.75) |
| Latin America and the Caribbean | - | **-0.06(-0.10, -0.02)** | 0.05(-0.21, 0.30) |
| Arab States | **1.92(1.53, 2.30)** | **-0.19(-0.30, -0.08)** | - |
| Sub-Saharan Africa | **2.74(2.15, 3.33)** | **-0.34(-0.53, -0.15)** | - |
| Asia | **4.08(2.45, 5.71)** | 0.05(-0.06, 0.16) | - |
| World | **2.73(2.51, 2.96)** | **-0.20(-0.22, -0.19)** | **-0.80(-0.98, -0.62)** |

| **Table S9** The effects of HDI changes (5, 50, and 95% percentiles) on TFR at lag0-5 years (reference = 0.67) | | | |
| --- | --- | --- | --- |
| Regions | Human development index | | |
|  | Low (0.37) | Median (0.70) | High (0.91) |
| Increase of fertility rate (95% CI) | | | |
| Western Europe and related countries | - | - | -0.18(-1.36, 1.00) |
| Eastern Europe | - | **-0.28(-0.36, -0.20)** | 0.13(-0.12, 0.38) |
| Latin America and the Caribbean | - | **-0.24(-0.31, -0.17)** | - |
| Arab States | **1.16(0.47, 1.86)** | **-0.34(-0.42, -0.25)** | - |
| Sub-Saharan Africa | **3.31(3.01, 3.60)** | **-0.42(-0.54, -0.29)** | - |
| Asia | **2.60(2.06, 3.14)** | **-0.29(-0.39, -0.19)** | **-1.68(-2.10, -1.26)** |
| World | **3.07(2.94, 3.20)** | **-0.28(-0.29, -0.26)** | **-0.93(-1.06, -0.79)** |


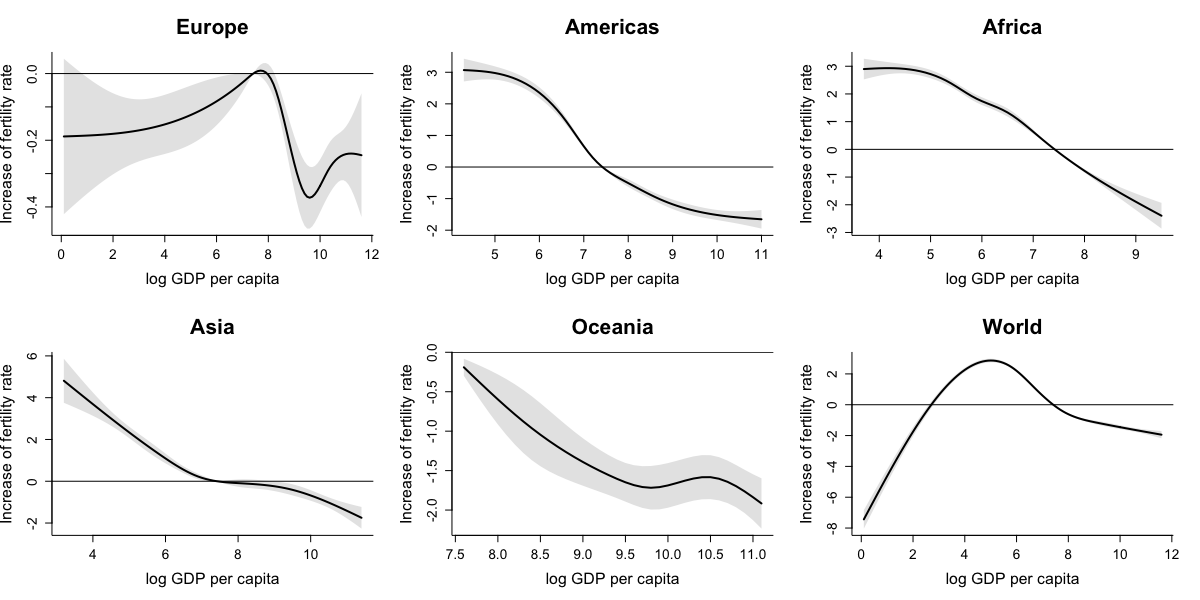


**Fig. S1** The estimated overall cumulative effects of mean log GDP per capita over 5 years on TFR with United Nations geographical divisions


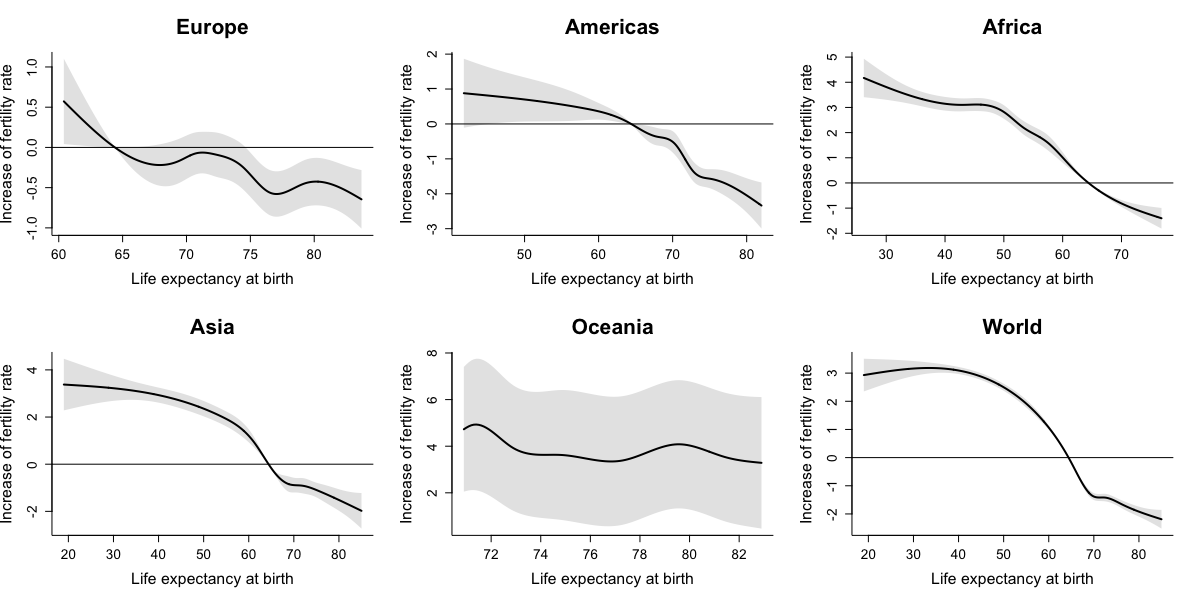


**Fig. S2** The estimated overall cumulative effects of mean life expectancy at birth over 5 years on total fertility rate (TFR) with United Nations geographical divisions


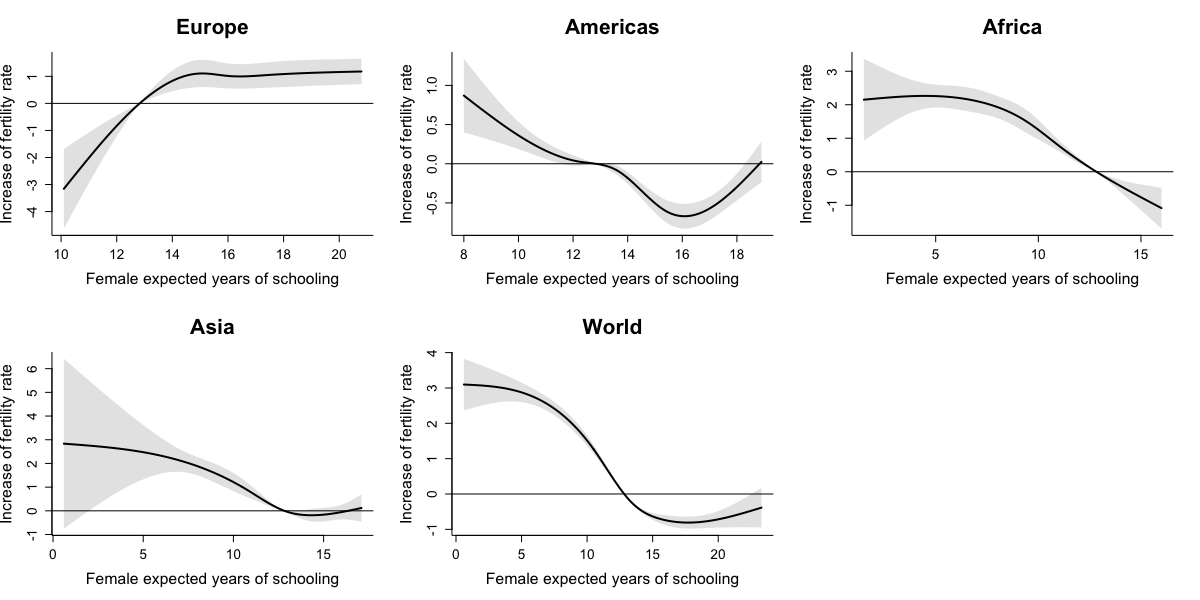


**Fig. S3** The estimated overall cumulative effects of mean female expected years of schooling over 5 years on TFR with United Nations geographical divisions


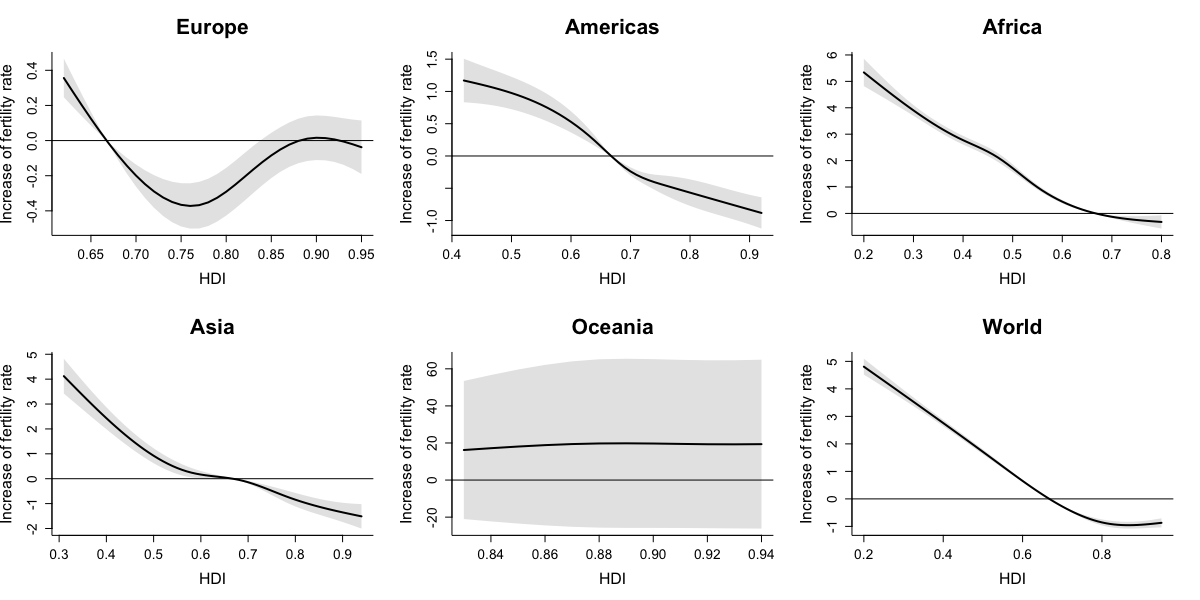


**Fig. S4** The estimated overall cumulative effects of mean human development index over 5 years on TFR with United Nations geographical divisions


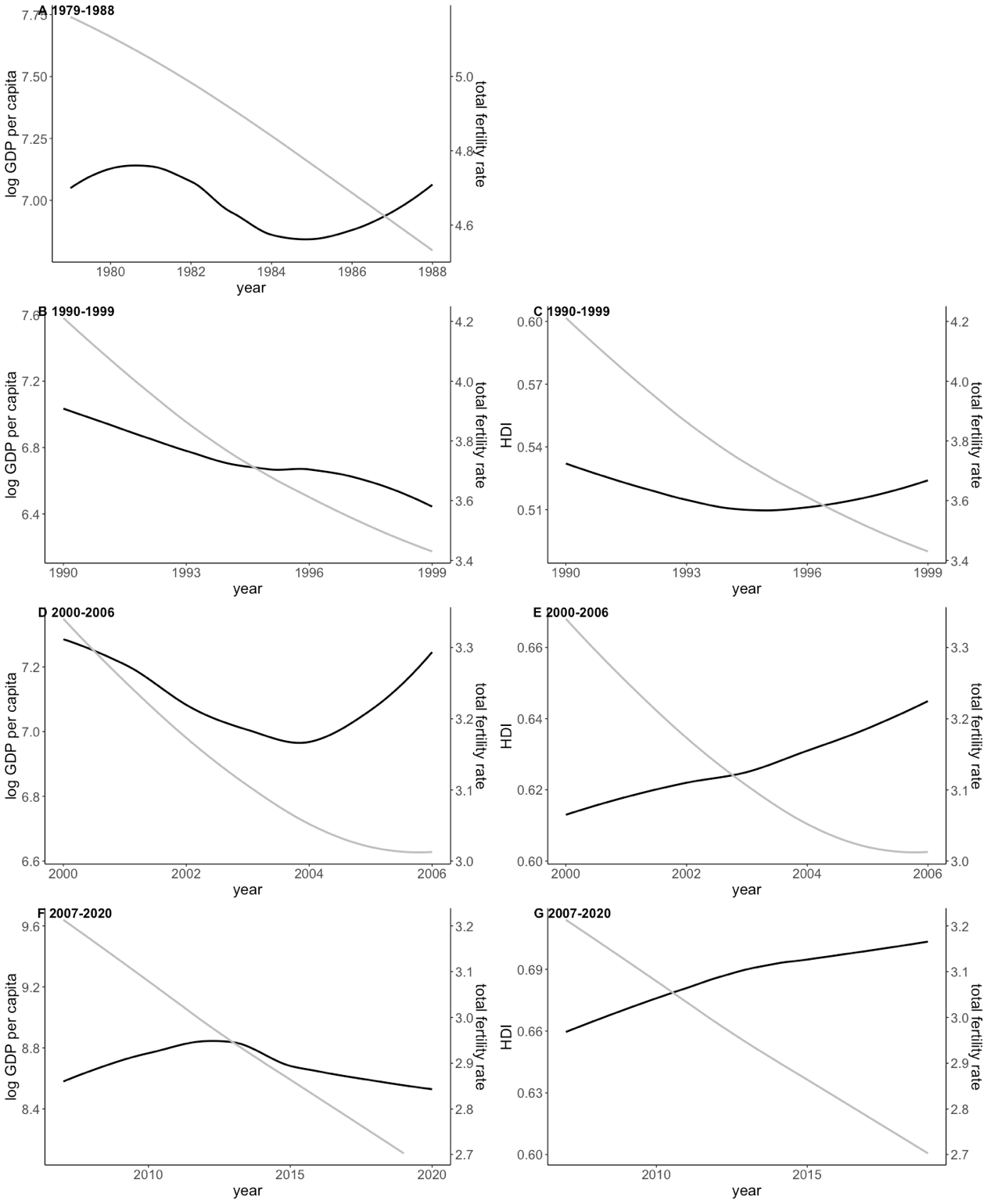


**Fig. S5** Trends of log GDP per capita (A, B, D, F), HDI (C, E, G) and TFR in economic recession countries during different periods


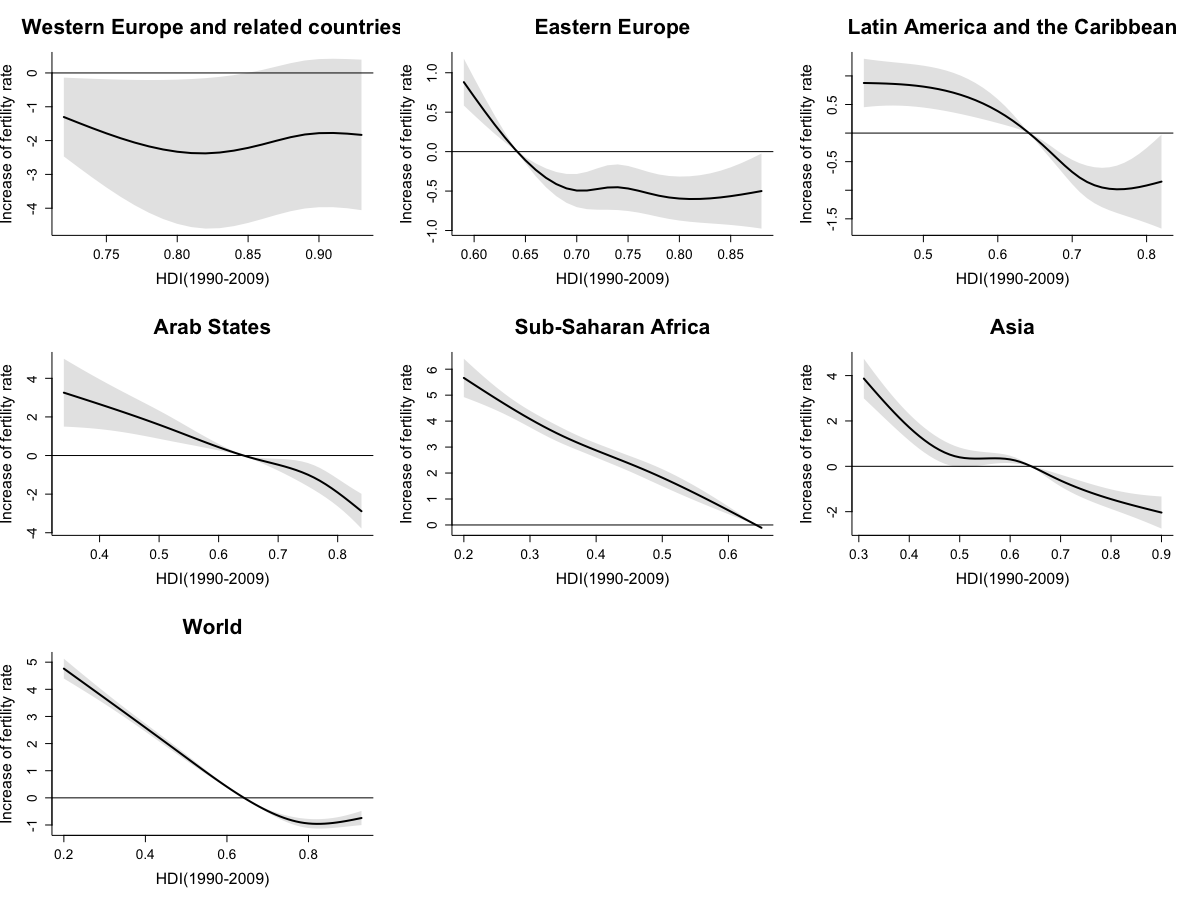


**Fig. S6** The estimated overall cumulative effects of mean human development index (1990-2009) over 5 years on TFR


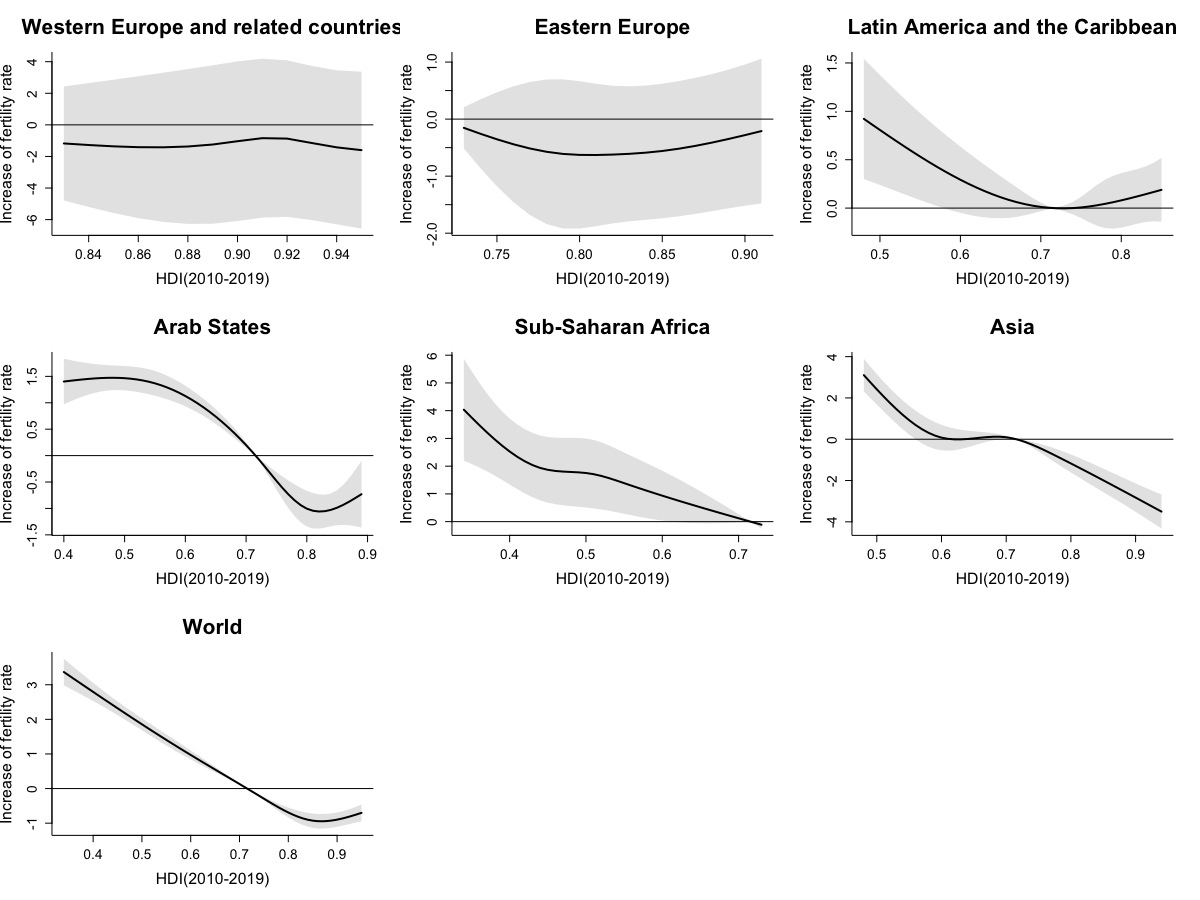


**Fig. S7** The estimated overall cumulative effects of mean human development index (2010-2019) over 5 years on TFR
